# The synergistic effects of organic composts and microelements co-application in enhancing potato productivity in saline soils

**DOI:** 10.1016/j.heliyon.2024.e32694

**Published:** 2024-06-12

**Authors:** Ayman M. El-Ghamry, Mohamed A. El-Sherpiny, Abd-Elbaset A. Alkharpotly, Dina A. Ghazi, Amal A. Helmy, Manzer H. Siddiqui, Mohammad Pessarakli, Mohammad Anwar Hossain, Eman M. Elghareeb

**Affiliations:** a Soil Sciences Department, Faculty of Agriculture, Mansoura University, 35516, Egypt; b Soil, Water and Environment Research Institute, Agriculture Research Center, El-Gama St., Giza, 12619, Egypt; c Horticulture Department, Faculty of Agriculture and Natural Resources, Aswan University, 81528, Egypt; d Horticulture Department, Faculty of desert and environmental agricultural, Matrouh University, 51511, Egypt; e Department of Botany and Microbiology, College of Science, King Saud University, Riyadh 11451, Saudi Arabia; f School of Plant Sciences, The University of Arizona, Tucson, AZ, USA; g Department of Genetics and Plant Breeding, Bangladesh Agricultural University, Mymensingh, 2202, Bangladesh; h Botany Department, Faculty of Science, Mansoura University, Mansoura, 35516, Egypt

**Keywords:** Antioxidants, Banana and soybean composts, Microelements, Salinity stress, Potato yield

## Abstract

Soil salinity is a major threat hindering the optimum growth, yield, and nutritional value of potato. The application of organic composts and micronutrients can effectively ameliorate the salinity-deleterious effects on potato growth and productivity. Herein, the combined effect of banana and soybean composts (BCo and SCo) application alongside foliar supplementation of boron (B), selenium (Se), cobalt (Co), and titanium (Ti) were investigated for improving growth, physiology, and agronomical attributes of potato plants grown in saline alluvial soil. Salinity stress significantly reduced biomass accumulation, chlorophyll content, NPK concentrations, yield attributes, and tuber quality, while inducing malondialdehyde and antioxidant enzymes. Co-application of either BCo or SCo with trace elements markedly alleviated salinity-adverse effects on potato growth and productivity. These promotive effects were also associated with a significant reduction in malondialdehyde content and activities of peroxidase and superoxide dismutase enzymes. The co-application of BCo and B/Se was the most effective among other treatments. Principle component analysis and heatmap also highlighted the efficacy of the co-application of organic composts and micronutrients in improving the salinity tolerance of potato plants. In essence, the co-application of BCo with B and Se can be adopted as a promising strategy for enhancing the productivity of potato crops in salt-affected soils.

## Highlights

• Salinity stress adversely affects potato growth, physiology, yield, and nutritional value.
• Co-application of banana compost with either boron or selenium is effective in improving potato yield and quality.
• The ameliorative effects of composts and microelement are associated with better antioxidant defense.

## Graphical abstract

**Figure:**
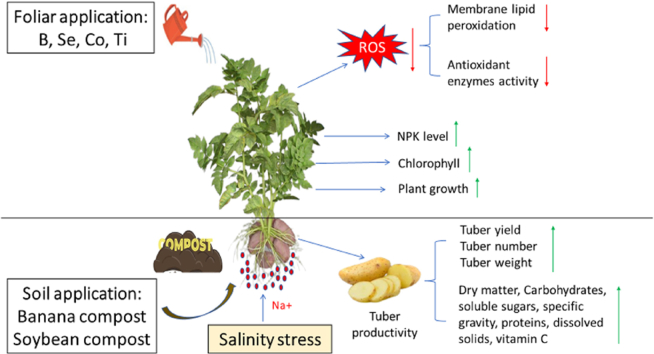

## 1 Introduction

Soil salinization is a critical and growing ecological threat that causes huge losses of arable soils [1], decreases soil fertility [2], and induces a sharp decline in crop productivity [3,4]. Approximately,840 million hectares of arable land (∼2 % of the total cultivated area worldwide) are negatively affected by soil salinity with 683 and 157 million hectares are classified as saline and saline-sodic soils, respectively [5]. In Egypt, arid and semi-arid area face significant challenges, where the soils are saline and characterized by limited rainfall, high evaporation rates, and elevated temperatures that certainly hinder agriculture sustainability development and food security [6,7]. Around 20–50 % of soils of the Nile Delta are categorized as salt-affected soils [8,9].Furthermore, the intensive anthropogenic activities and the ongoing global warming crisis are expected to intensify the soil salinization problem and increase the area of salt-impacted soils in Egypt [8]. The sharp productivity losses in saline soils and the overgrowth of the population put huge pressure on the agricultural sector to integrate sustainable, effective, and eco-friendly agricultural practices into crop fields to increase crops production under salinity stress. This will help to secure an adequate food supply for resource-poor individuals all over the globe. Soil salinity reduces crop productivity via various overlapping soil- and plant-related routes. It negatively impacts soil biochemistry by disrupting microbial communities, decreasing soil organic matter, reducing soil enzyme activities, and impairing plant nutrient availability and balance [10]. Salinity also induces ionic, osmotic, and severe oxidative stresses [[11], [12], [13]]. These stresses cause significant alterations in plant physiological processes, including nutrient uptake/assimilation and regular metabolic processes, that inhibit plant growth and development [14,15]. In response to soil salinity, many plants activate various cascades of intracellular physiological processes and metabolic adjustments to safeguard its hazardous effects. Examples include synthesizing and activating enzymatic/non-enzymatic antioxidants and the production of compatible osmoprotectants [16]. Such adaptive physiological responses are coordinately regulated to enable plants to scavenge the free radicals and minimize other salinity-induced harmful effects on plant growth and consequently enhance plant adaptation against salinity and improve crop yield [[17], [18], [19]].

In Egypt, within the intensive cropping systems, the agricultural residues crop and farm wastes could undergo composting rather than being burned to be used as a good organic fertilizer for crop cultivation [20]. Such organic amendment application has been used as an effective strategy to mitigate stressful environments, retain soil fertility, and boost crop yield, particularly by rural farmers, to overcome the high cost of synthetic fertilizers [21]. Examples include compost [22], vermicompost [23], biochar [24], plant growth-promoting rhizobacteria [25], humic substances [26,27], and bio-fertilizers [28]. Several strategies and management practices including the application of organic and inorganic amendments were implemented to lessen the negative impacts of salinity and increase the growth and productivity of several crops such as wheat, faba bean, barely, and fodder beet in Egypt [[29], [30], [31], [32]].Incorporating composts into saline soils during plowing could effectively revive poor soil with organic matter and various nutrients and improve the soil's physiochemical properties and biological activities [33]. Moreover, compost application could potentially modulate soil reaction, increase soil temperature, and maximize water and nutrient supply potentials of soil to reinforce the phyto-availability of nutrients and growth vigor of plants grown in salt-affected soils [34,35]. Organic manures also increase the soil's available carbon, decrease soil EC values, decrease soil Na^+^ and Cl^−^ ions, and enrich soil microbiome with beneficial salt-tolerant bacteria, which in turn improve soil characteristics [36,37].

Banana and soybean residues are valuable organic amendments with multifaceted benefits that can boost soil health and crop yields. The annual production of banana wastes approached 114.08 million metric tons, which poses environmental and economic concerns for banana farmers [38]. Likewise, huge amounts of waste are generated every year from soybean cultivation. For instance, 41 million tons/year of soybean waste are generated in Brazil alone [39]. The banana trees and soybean wastes are rich in valuable nutrients, vitamins, and hormones [[40], [41], [42]]. In fact, bananas wastes have an exceptional level of K (more than 10 %) among other agricultural wastes. Similarly, soybean composts are rich in essential nutrient content like N, P, and K [43]. Consequently, soil application of banana and/or soybean wastes as organic fertilizers might offer diverse approaches to improve soil physicochemical properties, water retention, and nutrient cycling, consequently boosting crop tolerance against biotic and abiotic stress conditions. However, the ameliorative effects of organic composts derived from banana trees and soybean residuals on salt-affected soils are not studied.

The effectiveness of organic amendments in enhancing crop production is significantly augmented by the combined application with microelements (non-essential elements for plants with high potential for promoting plant growth and development at very low concentrations). Examples of these elements include boron (B), selenium (Se), cobalt (Co), titanium (Ti), silicon (Si), aluminum (Al), and vanadium (V), which play relevant roles in mastering plant growth and resistance to abiotic stresses [[44], [45], [46]]. These elements reduce the harmful effects of salinity by inducing antioxidant systems, carbohydrate content, chlorophyll content, plant biomass, and crop yield. Trace elements could also enhance the utilization of other plant nutrients and nullify the hazardous effect of potentially toxic elements [[47], [48], [49]].Potato (*Solanum tuberosum* L.) is a staple food for most of the world's population, especially in Europe, America, and Egypt [20]. It is the fourth in human consumption after wheat, maize, and rice crops [50]. It markedly contributes to Egypt's export economy, playing a crucial role in boosting its income [51]. Potato is an inexpensive source of energy, minerals, vitamins, fibers, and organic acids [52,53]. According to Ref. [54], the Egyptian area cultivated with potatoes about 262.9 thousand hectares and the total production of tubers in the same year reached about 6.91 million tons. However, potatoes' growth and yield are significantly reduced by salinity, with up to 60 % loss in yield because of the salinity-induced reduction in tuberization [50]. Consequently, improving potato productivity in salt-affected soils can significantly contribute to the ongoing efforts to secure food supply for the increasing world population. In this regard, we hypothesize that applying banana and soybean composts, along with microelements, such as B, Se, Ti, and Co, can synergistically mitigate salinity stress and enhance the growth and productivity of potatoes cultivated in saline soils. Therefore, the main objective of the current study is to investigate the remediation potential of the combined application of organic manures and trace elements on the growth, yield, and underlying physiological mechanisms in potato (Cv Spunta) grown in a salt-affected soil.

## 2 Materials and methods

### 2.1 Materials

*Solanum tuberosum* L. Tubers (Cv Spunta) was obtained from the Egyptian Ministry of Agriculture and Soil Reclamation. Agricultural byproducts used for compost engineering (i.e., banana tree and soybean stover residuals) were obtained from private farms. The agricultural residuals were chopped into pieces (1–3 cm) and enriched with several supplementations to activate microorganisms and improve the nutrient content of compost: farmyard manure (1:5 w:w), ammonium sulfate (1:5 w:w), feldspar (1:100 w:w), and phosphate rock (1:100 w:w). The mixture was kept moistened at 60 % water holding capacity and the moisture content was compensated with water until a handful of mixtures would wet the hand but not drip. The mixture was turned once a week for aeration to accelerate the aerobic decomposition process until reaching the maturity stage (∼6.0 months). All chemical reagents of analytical grade, including calcium borate [Ca_3_ (BO_3_)_2_], sodium selenite [Na_2_SeO_3_], titanium dioxide [TiO_2_], and cobalt sulfate [CoSO_4_] were purchased from Sigma-Aldrich and used without further purification.

### 2.2 Study site

A field experiment was carried out in a private farm located at El-Serw village, El-Zarqa District, Damietta Governorate, Egypt (31°39′14″E longitude and 31°14′19″ N latitude) during the winter cropping season of 2022. The ecological zone is characterized by a moderate Mediterranean climate (the average precipitation values, minimum temperature, and maximum temperature during the growing season were 11 mm, 12.23 °C, and 18.85 °C, respectively). Damietta Governorate covers approximately 910 km^2^ in which the cultivated area comprises about 50 × 10^3^ ha. Soils in the region are classified into two main types: (i) agricultural non-saline soils of alluvial origin and (ii) barren saline soils originating from aeolian or marine sources.

### 2.3 Experimental design and treatments

The current field experiment was designed as a split-plot design with three replications in which compost amendments are the main factors and spraying of microelements is the subplot factor. Compost treatments include the following: control (no addition), banana compost (BCo), and soybean compost (SCo), while microelements sprays are: control (no spraying), boron (B), selenium (Se), cobalt (Co), and Titanium (Ti). Each plot area was 128 m^2^ (12.8 m × 10 m) with 15 rows (each row 85 cm × 10 m) and row spacing was 70 cm. Compost treatments were incorporated into the soil matrix one month before cultivation at a rate of 48.0 m^3^ ha^−1^. The soil underwent plowing to ensure the thorough mixing of compost materials after the incorporation. The tubers were hand-planted in 10–15 cm depth and spaced 20–30 cm with a density of about 44,000 plants ha^−1^. Superphosphate (15 % P_2_O_5_) was added at a rate of 240 kg ha^−1^ during soil preparation. Nitrogen fertilizer was applied at the rate of 300 kg N ha^−1^ in the form of ammonium sulfate (21 % N) in two equal doses at 30 and 60 days from planting. Potassium sulfate (48 % K_2_O) was added at 60 days from planting at a rate of 240 kg K ha^−1^. Two days before planting, potato tubers were divided into pieces with an average weight of 45.0 g and planted in wet soil on the 15th of January. Potato plants were sprayed with the tested microelements (B, Se, Ti, and Co) at concentrations of 20.0 mg L^−1^ immediately after the first nitrogen dose and repeated three times at 20-day intervals. The other recommended agricultural practices and irrigation processes were carried out according to traditional recommendations for potato production.

### 2.4 Soil and compost analysis

The soil sample at a depth of 0–30 cm was collected before cultivation, air-dried to a constant weight, crushed using a wooden pestle, and sieved through a 2.0 mm sieve. Physical and chemical analyses of soil were performed according to Ref. [55] and presented in Table 1. Composite compost samples were also collected from several spots of the pile to conduct the routine analysis [56]. The chemical composition of compost samples is presented in Table 2. The pH in the compost suspension (1:10) was measured using a pH meter and the electric conductivity (EC) in the suspension was estimated using an EC meter (HANNA Instrument, HI 8033). The total nitrogen (N) was determined Kjeldahl method as described by Ref. [57], while the total carbon was measured following the method of [58]. The phosphorus (P) and manganese (Mn) were determined according to Ref. [59]. A flame photometer (PFP7, Jenway company, Türkiye) was used for the determination of K content.

**Table 1:** Physical and chemical properties of experimental soil before cultivation.

Soil properties	Values
**Sand (%)**	25.89
**Silt (%)**	23.6
**Clay**	50.51
**Soil texture**	Clay
**Saturation (%)**	92
**Field capacity (%)**	46
**Wilting point (%)**	23
**pH**	7.92
**Electric conductivity (dSm**^**−**^**^1^)**	5.79
**Organic matter (%)**	1.09
**Available nitrogen (mgkg**^**−**^**^1^)**	47
**Available phosphorus (mgkg**^**−**^**^1^)**	8.99
**Available potassium (mgkg**^**−**^**^1^)**	225.1

**Table 2:** Some chemical properties of banana and soybean stover composts.

Chemical properties	Banana tree residuals compost	Soybean stover compost
pH	6.25	6.44
Electric conductivity (dSm^−1^)	3.50	3.55
Total carbon (%)	19.2	19.8
Total nitrogen (%)	1.70	1.70
Carbon:Nitrogen ratio	11.3	11.65
Phosphorus (mg kg^−1^)	0.72	0.59
Potassium (%)	4.95	0.77
Manganese (mg kg^−1^)	23.0	24.0

### 2.5 Vegetative growth parameters, yield, and yield-related traits

At 75 days after planting, vegetative growth parameters of three plants including fresh weight and plant height were recorded. Afterward, plant samples were oven-dried at 70 °C until constant weights. The dry weight was recorded, and the oven-dried samples were ground into a homogenous fine powder using an electric stainless-steel grinder and used for downstream biochemical analyses. At the harvesting stage (120 days after planting), the total tuber yield (metric ton h^−1^) was determined. Thirty plants were randomly selected and the total number of tuber plant^−1^ was recorded. Thirty representative potato tubers from each treatment were selected and the average tuber weight (g) was recorded. The nutritional contents of potato tubers, including total carbohydrates, total sugars, dry matter, vitamin C, total dissolved solids (TDS), total proteins, and specific gravity were quantified according to the methods of [60].

### 2.6 Chlorophyll content and nutrient concentrations

Total chlorophyll content in the leaves of three representative plants was measured between 10 and 12 a.m. with a portable chlorophyll meter (SPAD-502, Minolta Camera, Osaka, Japan) following the procedures suggested by Ref. [61]. Plant nutrients, including N, P, and K were determined in the acid-digestion (H_2_SO_4_/HClO_4_) of dry powdered leaf tissues using the protocol of [62]. Total N concentration was determined using the Kjeldahl method, total P was calorimetrically (Spectromax-M5- USA) determined using the ascorbic acid method [63], and total K concentration was determined using the flame photometric method(PFP7, Jenway company, Türkiye).

### 2.7 Malondialdehyde concentration and antioxidant enzyme activities

Fresh leaves of three plants at 75 days after planting were instantly frozen in liquid N, saved at −80 °C, and used for the analysis of malondialdehyde (MDA), and the activities of antioxidant enzymes. MDA was measured using the modified protocol of [64]. First, about 1.0 g of frozen leaf tissues were ground with 10 ml of 10 % trichloroacetic (TCA) and then centrifuged at 5000 g for 10 min. Subsequently, 2.0 ml of the supernatant was mixed with 2.0 ml of thiobarbituric acid (TBA), mixed well, and kept in a boiling water bath for 15 min. The reaction mixtures were cooled down on the ice for 10 min and the absorbance of the developed color was measured at 520 and 600 nm using a spectrophotometer (Spectromax-M5- USA). The amount of MDA was calculated by subtracting absorbance at 600 nm from that at 532 nm, corrected by an extinction coefficient 155 nM^−1^cm^−1^, and expressed as μmolg^−1^ FWT.

For antioxidant enzymes, known weights of frozen leaf tissues were homogenized in 5 mL extraction buffer (1.0 M phosphate, 0.5 mM EDTA, pH 7.0). The homogenates were centrifuged at 10,000 rpm for 15 min at 4 °C, and supernatants were collected and raised to a known volume and used to determine enzyme activities. Peroxidase enzyme (POD) was determined by the procedures adopted by Ref. [65]. The reaction mixture contained 100 ml of 0.05 M potassium phosphate buffer (pH 7.8), 10 ml H_2_O_2_ (0.3 %), 10 ml guaiacol (1 %), and 0.1 ml enzyme extract. The absorbance change was determined at 470 nm. One unit of POD was expressed as the absorbance change of 0.01 in 1 min. Superoxide dismutase (SOD) activity was measured spectrophotometrically (Spectromax-M5- USA) based on decreasing in superoxide-nitro blue tetrazolium complex (NBT) at 560 nm [66]. The reaction mixture consists of 0.05 ml of the crude enzyme, 0.1 ml of 1.5 M sodium carbonate, 0.2 ml of 200 mM methionine, 0.1 ml of 2.25 mM Nitro blue tetrazolium, 0.1 ml of 3 mM EDTA, 1.5 ml of 100 mM potassium phosphate buffer, 0.1 ml riboflavin (60 mM), and 1.0 ml distilled water. The sample tubes were illuminated at a light intensity of 15 W for 15 min. One SOD unit was expressed as the amount of the enzyme that reduced 50 % of NBT was recorded.

### 2.8 Statistical analysis

Data were statistically analyzed using the CoStat software package (Version 6.303, CoHort, USA, 1998–2004). Treatment means were compared by using the least significant difference (LSD) test at a 0.05 level of probability. PCA was conducted by Origin 9 and a heatmap for the plant growth traits and the physiochemical properties of treated potato tubers were performed using GraphPad Prism 9.

## 3 Results and discussion

### 3.1 Effects of composts and microelements application on growth traits of salt-stressed potato plants

Data in Table 3 revealed that the growth traits of potato plants were significantly influenced (P < 0.05) by the addition of organic composts and microelements either individually or in combination. Throughout the experiment, the salinity-stressed potato exhibited the lowest growth indices including plant height, fresh weight, and dry weight, indicating the suppressive effects of salinity on the growth of potato plants. Conversely, compost treatments, trace elements application, and their interactions all showed promotive effects on the vegetative growth indices of salt-stressed potato plants. Compared to control treatments, the application of BCo or SCo enhanced all growth indices; however, the former was consistently more effective than the latter. Also, potato plants treated with the tested micronutrients had better growth than their control peers, in which B and Se showed higher promotive effects than Co and Ti. Further, the combined application of BCo with B elicited the highest increase in plant height (20.30 %), fresh (25 %), and dry weights (17.29 %) during the growing season.

**Table 3:** Effect of adding organic manures, microelements, and their interaction on growth performance of potato plants grown on salt-affected soil for 75 days from planting.

		Plant height (cm)	Fresh weight (g)	Dry weight (g)
Compost manures (Co)				
Control		47.24c ± 0.26	197.76c ± 0.98	42.60c ± 0.25
Banana (BCo)		54.01a ±0.35	231.68a ±3.10	46.83a ±0.34
Soybean(SCo)		52.23b ± 0.30	219.26b ± 1.87	45.49b ± 0.20
Foliar application Control (C)		49.1e ± 0.84	203.72e ± 2.51	43.24e ± 0.55
Boron (B)		52.28a ±1.02	225.02a ±5.38	46.03a ±0.62
Selenium (Se)		51.98b ± 1.00	220.45b ± 6.11	45.63b ± 0.62
Cobalt (Co)		51.65c ± 1.02	217.21c ± 5.50	45.27c ± 0.67
Titanium (Ti)		50.72d ± 1.16	214.76d ± 5.25	44.70d ± 0.67
Interaction				
Control	**C**	45.90o ± 0.05	193.75o ± 0.22	41.00 m ± 0.089
	**B**	48.34k ± 0.06	204.57k ± 0.18	43.75i ± 0.058
	**Se**	48.07l ± 0.039	198.16l ± 0.27	43.27j ± 0.026
	**Co**	47.68 m ± 0.046	196.58 m ± 0.15	42.71k ± 0.053
	**Ti**	46.23n ± 0.055	195.74n ± 0.19	42.26l ± 0.036
BCo	**C**	51.49i ± 0.034	209.71i ± 0.25	44.37h ± 0.10
	**B**	55.22a ±0.059	242.13a ±0.18	48.09a ±0.053
	**Se**	54.76b ± 0.074	240.35b ± 0.32	47.49b ± 0.091
	**Co**	54.53c ± 0.052	234.20c ± 0.32	47.27c ± 0.087
	**Ti**	54.07d ± 0.044	232.00d ± 0.21	46.94d ± 0.087
SCo	**C**	50.13j ± 0.071	209.71i ± 0.20	44.37h ± 0.04
	**B**	53.29e ± 0.04	228.35e ± 0.32	46.25e ± 0.052
	**Se**	53.12f ± 0.07	222.84f ± 0.20	46.14e ± 0.040
	**Co**	52.75g ± 0.049	220.87g ± 0.29	45.84f ± 0.055
	**Ti**	51.857h ± 0.063	216.56h ± 0.24	44.91g ± 0.018

Data represent the mean of three biological replicates ± SE. Means within a column followed by a different letter (s) are statistically different at a 0.05 probability level.

The inhibitory effects of salinity on the growth of potato plants are primarily attributed to its osmotic stress-induced ion toxicity that detrimentally influences plant hormones, cell division, and differentiation, hampering overall plant growth [[67], [68], [69]]. On the other hand, the notable effectiveness of BCo and SCo, either alone or in combination with the micronutrients in enhancing potato growth align with numerous previous studies that highlight the potential of comparable amendments to improve salinity resistance [[70], [71], [72]]. Similar results for the effect of BCo in improving the fresh and dry mass of barely under Cd stress were reported by Ref. [31]. The promotive effects of these organic manures are based on their ability to enrich soil nutrient availability and uptake as well as soil organic matter and biological activity [73,74]. Meanwhile, the superiority of BCo over SCo may be attributed to its high content of essential K nutrient (Table 3) which counteract the negative effects of sodium ions in saline soils [36,75]. 10.13039/100014337Furthermore, our results well support the reported safeguard effects of exogenous B, Se, and Co application in ameliorating the adverse impacts of salinity [[76], [77], [78]]. Indeed, the B additives to banana-composted plants enhance various physiological processes crucial for plant growth and yield such as cell elongation and maturation, protein synthesis, development of meristematic tissues, integrity of plasma membrane, and cell wall [[76], [77], [78]]. These mechanisms collectively suggest the potential of BCo enriched with B as a sustainable approach for improving potato cultivation in saline soils, offering practical solutions for agricultural challenges.

### 3.2 Effect of composts and microelements application on tubers yields of salt-stressed potato plants

Among treatments, salinity-grown potato plants had the lowest tuber yield, tuber number, and tuber weight (Table 4). The application of BCo and SCo, either alone or combined with trace elements, induced pronounced changes in the tubers' yield components of potato plants. Compared to control plants, BCo stimulated the average tuber weight and yield, while SCo positively increased the number of tubers per plant. In addition, all foliar treatments substantially boosted tuber yields and ranked as B > Se > Co > Ti, respectively. The highest tuber weight and yield were obtained by the combined application of BCo and B; meanwhile, SCo, along with either B or Se resulted in the highest number of plant tubers. Salinity stress caused disorders in potato growth and biomass accumulation which directly reduced the tuber yield of potato plants. On the other hand, the increase in potato tuber characteristics with the co-application of organic composts and tested elements might be due to the compost's role in fostering soil organic matter which ultimately stimulates the release of essential nutrients inherent to soil (N, P, K), addressing deficiencies in saline-affected soils [36]. Such a combination could also alter the plant ionic balance and increase the supply of leaf NPK, which are linked to increasing chlorophyll content and photosynthetic activity. These advancements, in turn, boost the antioxidant system, growth, and dry matter accumulation, resulting in yield penalty. Indeed, substantial K content in BCoplays a pivotal role in the enhancing productivity of potato tubers by regulating of opening and closing of stomata and increasing photosynthesis, nutrient translocation, water uptake, and the final tuber yield [79].

**Table 4:** Effect of adding organic manures, microelements, and their interaction on tubers yield of potato plants grown on salt-affected soil for 120 days from planting.

		Average tuber weight (g)	Number of tubers plant^-1^	Yield (Metric ton h^-1^)
Compost manures (Co)				
Control		123.95c ± 0.15	4.52c ± 0.04	12.33c ± 0.13
Banana (BCo)		143.21a ±2.04	4.89b ± 0.005	15.42a ±0.22
Soybean (SCo)		133.61b ± 1.09	4.91a ± 0.014	14.45b ± 0.15
Foliar applications				
Control (C)		126.50e ± 0.84	4.64e ± 0.097	12.94e ± 0.35
Boron (B)		138.28a ± 3.86	4.85a ± 0.030	14.79a ± 0.47
Selenium (Se)		136.05b ± 3.36	4.83b ± 0.049	14.50b ± 0.47
Cobalt (Co)		134.47c ± 3.05	4.79c ± 0.061	14.21c ± 0.48
Titanium (Ti)		132.64d ± 2.84	4.75d ± 0.082	13.89d ± 0.49
Interaction				
Control	**C**	123.34l ± 0.28	4.25k ± 0.008	11.55o ± 0.013
	**B**	124.51k ± 0.33	4.74g ± 0.004	12.99k ± 0.028
	**Se**	124.28k ± 0.19	4.64h ± 0.005	12.70l ± 0.020
	**Co**	123.91 kl ± 0.24	4.54i ± 0.005	12.39 m ± 0.012
	**Ti**	123.70 kl ± 0.30	4.41j ± 0.003	12.02n ± 0.025
BCo	**C**	128.94i ± 0.17	4.86e ± 0.005	13.80i ± 0.025
	**B**	151.26a ± 0.33	4.88de ± 0.005	16.24a ± 0.027
	**Se**	147.57b ± 0.36	4.90bc ± 0.004	15.93b ± 0.032
	**Co**	145.09c ± 0.27	4.91bc ± 0.009	15.70c ± 0.020
	**Ti**	143.18d ± 0.23	4.90cd ± 0.006	15.44d ± 0.013
SCo	**C**	127.24j ± 0.35	4.81f ± 0.011	13.46j ± 0.019
	**B**	139.07e ± 0.17	4.95a ± 0.006	15.15e ± 0.012
	**Se**	136.29f ± 0.36	4.96a ± 0.010	14.88f ± 0.018
	**Co**	134.42g ± 0.25	4.91bc ± 0.006	14.54g ± 0.013
	**Ti**	131.05h ± 0.25	4.92b ± 0.009	14.21h ± 0.017

Data represent the mean of three biological replicates ± SE.Means within a column followed by a different letter (s) are statistically different at 0.05 probability level.

Furthermore, the tested microelements induce the synthesis of plant hormones that promote plant growth and yield [80]. Similar results were reported by Refs. [20,72] in potato. These findings validate the fulfillment of our hypotheses regarding the importance of biofertilization of saline soil with composts along with micronutrients as a promising and effective agricultural strategy to bolster potato yield in saline agricultural soils.

### 3.3 Effect of composts and microelements application on tubers quality of salinity-stressed potato

Results in Table 5, Table 6 demonstrated a significant enhancement in nutritional quality traits of potatoes (total carbohydrates, total sugars, dry matter, vitamin C, specific gravity, TDS, and protein) by application of tested composts and micronutrients compared to the control plants. BCo induced the maximum average values of all the investigated traits.

**Table 5:** Effect of adding organic manures, microelements, and their interaction on tubers quality traits (total carbohydrates, total sugars, dry matter, and vitamin C) of potato grown on salt-affected soil for 120 days from planting.

		Total carbohydrates (%)	Total sugars (%)	Dry matter (%)	Vitamin C (mg100g^−1^)
Compost manures (Co)					
Control		24.24c ± 0.072	4.75c ± 0.023	18.03c ± 0.077	19.47c ± 0.057
Banana (BCo)		26.14a ±0.16	5.28a ±0.040	19.96a ±0.160	21.17a ±0.199
Soybean (SCo)		25.46b ± 0.109	5.09b ± 0.022	19.17b ± 0.103	20.33b ± 0.088
Foliar applications					
Control (C)		24.54e ± 0.18	4.87d ± 0.055	18.34d ± 0.18	19.65d ± 0.11
Boron (B)		25.76a ±0.31	5.18a ±0.077	19.48a ±0.31	20.88a ±0.35
Selenium (Se)		25.57b ± 0.32	5.09b ± 0.084	19.30 ab ± 0.31	20.50b ± 0.27
Cobalt (Co)		25.36c ± 0.30	5.05c ± 0.086	19.13bc ± 0.31	20.37bc ± 0.26
Titanium (Ti)		25.16d ± 0.27	5.02c ± 0.085	19.03c ± 0.30	20.23c ± 0.26
Interaction					
Control	**C**	23.80 m ± 0.27	4.65k ± 0.018	17.66h ± 0.127	19.24h ± 0.083
	**B**	24.62j ± 0.023	4.90h ± 0.018	18.38 fg ± 0.125	19.75fgh ± 0.80
	**Se**	24.36k ± 0.033	4.78h ± 0.017	18.11gh ± 0.120	19.53gh ± 0.129
	**Co**	24.24 kl ± 0.018	4.73hi ± 0.018	18.02gh ± 0.128	19.47gh ± 0.071
	**Ti**	24.16l ± 0.024	4.70hi ± 0.017	17.97gh ± 0.124	19.39gh ± 0.083
BCo	**C**	25.02h ± 0.030	4.99f ± 0.020	18.85def ± 0.122	19.91efg ± 0.136
	**B**	26.76a ±0.020	5.42a ±0.020	20.49a ±0.138	22.16a ±0.145
	**Se**	26.55b ± 0.036	5.36 ab ± 0.023	20.24a ±0.158	21.42b ± 0.076
	**Co**	26.31c ± 0.036	5.33abc ± 0.021	20.21a ±0.073	21.24bc ± 0.085
	**Ti**	26.07d ±0.024	5.28b ± 0.018	20.03 ab ± 0.086	21.14bc ± 0.139
SCo	**C**	24.81i ± 0.024	4.96g ± 0.018	18.51 fg ± 0.075	19.80 fg ± 0.071
	**B**	25.92e ±0.036	5.23c ± 0.020	19.57bc ± 0.080	20.72cd ± 0.083
	**Se**	25.80e ± 0.024	5.14d ± 0.020	19.54bc ± 0.087	20.55d ± 0.075
	**Co**	25.54f ± 0.035	5.08ef ± 0.020	19.18cd ± 0.069	20.41de ± 0.150
	**Ti**	25.24g ± 0.028	5.06ef ± 0.018	19.08cde ±0.130	20.17def ± 0.130

Data represent the mean of three biological replicates ± SE. Means within a column followed by a different letter (s) are statistically different at 0.05 probability level.

**Table 6:** Effect of adding organic manures, microelements, and their interaction on tubers quality traits (specific gravity, TDS, and protein) of potato grown on salt-affected soil for 120 days from planting.

		Specific gravity (%)	Total dissolvedsolids (%)	Protein (%)
Compost manures (Co)				
Control		1.03c ± 0.001	5.76c ± 0.036	11.50c ± 0.038
Banana (BCo)		1.06a ±0.002	6.93a ±0.092	13.60a ±0.181
Soybean (SCo)		1.05b ± 0.001	6.52b ± 0.056	12.89b ± 0.171
Foliar applications				
Control(C)		1.04d ± 0.003	5.99d ± 0.117	11.78d ± 0.142
Boron (B)		1.059a ±0.004	6.63a ±0.189	13.14a ±0.371
Selenium (Se)		1.055 ab ± 0.004	6.56a ±0.186	12.94b ± 0.358
Cobalt (Co)		1.053bc ± 0.004	6.48b ± 0.196	12.74c ± 0.346
Titanium (Ti)		1.051c ± 0.004	6.36c ± 0.179	12.72c ± 0.350
Interaction				
Control	**C**	1.03j ± 0.001	5.53l ± 0.024	11.29i ± 0.049
	**B**	1.04g ± 0.0001	5.91i ± 0.024	11.67 fg ± 0.049
	**Se**	1.04gh ± 0.001	5.86ij ± 0.021	11.57gh ± 0.047
	**Co**	1.03hi ± 0.002	5.780jk ± 0.024	11.52h ± 0.043
	**Ti**	1.03ij ± 0.001	5.72k ± 0.023	11.48h ± 0.049
BCo	**C**	1.05f ± 0.003	6.27g ± 0.023	12.26e ± 0.052
	**B**	1.07a ±0.001	7.18a ±0.029	13.99a ±0.054
	**Se**	1.06 ab ± 0.001	7.13a ±0.031	13.96a ±0.050
	**Co**	1.06 ab ± 0.002	7.13a ±0.068	13.91 ab ± 0.054
	**Ti**	1.06bc ± 0.0001	6.95b ± 0.030	13.90 ab ± 0.054
SCo	**C**	1.05f ± 0.001	6.17h ± 0.026	11.80f ± 0.041
	**B**	1.06bc ± 0.001	6.80c ± 0.028	13.77b ± 0.053
	**Se**	1.06cd ± 0.00	6.68d ± 0026	13.31c ± 0.049
	**Co**	1.05de ± 0.002	6.54e ± 0.023	12.79d ± 0.054
	**Ti**	1.054ef ± 0.001	6.42f ± 0.026	12.78d ± 0.047

Data represent the mean of three biological replicates ± SE. Means within a column followed by a different letter (s) are statistically different at a 0.05 probability level.

Furthermore, foliar B application enhanced total carbohydrates, sugars, vitamin C, and protein. It was statistically at par with Se for the higher dry matter, specific gravity, and TDS compared to the other elements. The combined application of BCo, either with B or Se or Co, induced the greatest improvement in tubers quality compared to other treatments.

Reductions in tuber's chemical compositions of salinity-stressed potatoes are related to the negative effects of salinity on growth, chlorophyll content, and nutrient uptake of plants, resulting in a final reduction in tuber quality [14]. On the contrary, the positive effects of BCo and the elemental supplement on the quality of potato tubers may be attributed to their role in intensifying translocation and conversion of photosynthetases from source leaves into starch, protein, and vitamins in sink tubers, which finally increased total carbohydrates, total sugars, and proteins of potato tubers [81]. The high level of K in BCo also might increase dry matter accumulation and starch via activating starch synthase enzyme in potato tubers [82]. The final increment in dry matter of potato tubers by organic manures and trace elements is essential for achieving the desired crispy texture in potato chips [83]. Similar findings for the positive effects of organic manures on tubers quality have been reported [20,84]. Thus, the current results confirm that the integrated application of banana and soybean composts with micronutrients are promising management practices that might provide an alternative approach to increase the quantity and quality of potato plants in saline environments.

### 3.4 Effect of composts and microelements application on chlorophyll content and nutrients status of salt-stressed potato plants

As shown in Table 7, salinity stress caused a substantial decline in chlorophyll content and leaf N, P, and K concentrations compared to control plants. The application of either BCo or SCo significantly reversed this trend and increased the content of chlorophyll as well as N, P, and K in potato leaves. Further, BCo application resulted in a greater enhancement of chlorophyll and these vital nutrients compared to SCo, suggesting its efficiency in mitigating salinity-induced nutrient depletion. Co-treatments of either of the two composts with each of the tested elements significantly enhanced the chlorophyll and essential leaf N, P, and K content of salinity-stressed potato plants compared to the compost alone. Notably, BCo co-applying with B and Se induced chlorophyll content and the higher accumulation in N, P, and K outperforming other elements like Co and Ti throughout the cropping season.

**Table 7:** Effect of adding organic manures, microelements, and their interaction on chlorophyll content and nutrient composition in leaves of potato plants grown on salt-affected soil for 75 days from planting.

		Chlorophyll, SPAD reading	Nitrogen (%)	Potassium (%)	Phosphorus (%)
Compost manures(Co)					
Control		38.85c ± 0.07	2.73c ± 0.024	0.318c ± 0.002	2.89c ± 0.025
Banana (BCo)		40.23a ±0.12	3.27a ±0.040	0.380a ±0.004	3.24a ±0.028
Soybean (SCo)		39.69b ± 0.07	3.05b ± 0.023	0.360b ± 0.002	3.11b ± 0.018
Foliar application					
Control (C)		39.06e ± 0.14	2.85d ± 0.061	0.333e ± 0.006	2.92e ± 0.052
Boron (B)		39.98a ±0.22	3.15a ±0.081	0.369a ±0.010	3.17a ±0.057
Selenium (Se)		39.82b ± 0.20	3.07b ± 0.082	0.358b ± 0.009	3.13b ± 0.060
Cobalt (Co)		39.63c ± 0.21	3.03b ± 0.088	0.355c ± 0.009	3.07c ± 0.068
Titanium (Ti)		39.47d ± 0.23	2.97c ± 0.085	0.349d ± 0.009	3.02d ± 0.071
Interaction					
Control	**C**	38.507l ± 0.086	2.61g ± 0.024	0.306l ± 0.001	2.71k ± 0.010
	**B**	39.16j ± 0.044	2.85e ± 0.025	0.330h ± 0.001	2.96h ± 0.011
	**Se**	39.10j ± 0.060	2.80e ± 0.015	0.323i ± 0.001	2.90i ± 0.011
	**Co**	38.86k ± 0.038	2.73f ± 0.025	0.319j ± 0.001	2.81j ± 0.011
	**Ti**	38.63l ± 0.050	2.67 fg ± 0.025	0.313k ± 0.001	2.74k ± 0.010
BCo	**C**	39.41hi ± 0.073	2.99cd ± 0.028	0.348g ± 0.001	3.05f ± 0.014
	**B**	40.73a ±0.068	3.40a ±0.02	0.405a ±0.001	3.35a ±0.011
	**Se**	40.50b ± 0.047	3.36a ±0.032	0.386b ± 0.001	3.32a ±0.013
	**Co**	40.34bc ± 0.09	3.33a ±0.029	0.382bc ± 0.001	3.27b ± 0.019
	**Ti**	40.20cd ± 0.080	3.25b ± 0.019	0.381c ± 0.001	3.23c ± 0.011
SCo	**C**	39.41hi ± 0.069	2.94d ± 0.028	0.345g ± 0.001	2.99g ± 0.011
	**B**	40.05de ± 0.030	3.19b ± 0.029	0.372d ± 0.001	3.19cd ± 0.011
	**Se**	39.86ef ± 0.081	3.06c ± 0.030	0.367e ± 0.001	3.17d ± 0.013
	**Co**	39.69 fg ± 0.079	3.05c ± 0.017	0.364e ± 0.001	3.13e ± 0.013

Data represent the mean of three biological replicates ± SE. Means within a column followed by a different letter (s) are statistically different at a 0.05 probability level.

The obtained detrimental impacts of salinity on chlorophyll content are consistent with the reported decline in photosynthetic pigments in salinity-stressed rice [85], tomato [86], and eggplant [15]. The salinity stress suppressed chlorophyll content resulting in disrupting photosynthesis and chloroplast ultrastructure [87]. Additionally, it reduces the precursors necessary for chlorophyll biosynthesis and in the meantime induces chlorophyll degradation via activation of chlorophyllase activity [88]. Further, salinity induces the accumulation of toxic levels of Na^+^ ions and hinders the absorption of N and Mg elements, which are vital elements for chlorophyll synthesis and photosynthesis [69]. The induced accumulation of chlorophyll in potato leaves in response to the application of BCo and B most likely occurs as a result of their vital role in enriching soil fertility, enhancing soil nutrient availability, and improving N, P, and K uptake by potato plants [89,90]. Furthermore, humic substances derived from organic compost application might inhibit the activity of chlorophyllase enzyme and thus delay chlorophyll senescence in plants [91].

In addition, the salinity-induced imbalance in the absorption, transportation, and utilization of essential nutrients, as well as the disruption of the water status in plants, are among the main deteriorative effects of salinity on plant growth and development. The BCo-induced increments in leaf N, P, and K content reflect its crucial role in stimulating the availability of these essential nutrients in the soil and their uptake by potato plants in salt-affected soils. Similar positive effects of organic compost on NPK concentration in plant leaves were previously reported in potatoes [68], kiwifruit [90], and strawberry [92]. The application of composts increases the nutrient status of potatoes by reducing the high soil alkalinity and increasing soil cation exchange capacity, thereby encounters the salinity-induced ion imbalance. In addition, organic manures enhance soil microbial activity, resulting in nutrient solubilization and subsequent availability and uptake. The notable enhancement in yield characteristics of potato tubers upon the application of BCo alongside B and Se can be linked to their role in increasing essential leaf nutrients that fortified translocation of photo-assimilates from the source leaves to the sink tubers and, consequently, improving tubers yield [93]. The current findings are consistent with those of [67,94]. The additive enhancement in leaf NPK of potato-stressed plants in response to the co-treatment of either of the two composts with the microelements coincides with their reported role in increasing the proliferation of plant root systems [95].

### 3.5 Effect of composts and microelements application on MDA and antioxidants enzymes in salt-stressed potato plants

The salinity-stressed potato plants exhibited the highest increase in foliar MDA levels as well as POD and SOD enzyme activities (Table 8). Amending saline soil with BCo and SCo reduced the oxidative stress markers in potatoes, including MDA content and activities of POD and SOD enzymes compared to their respective controls. Compared to salinity-stressed plants, BCo induced about a 28.25 % reduction in MDA, whereas the corresponding reductions in POD and SOD approached 28.89 % and 19.87 %, respectively. Similarly, SCo reduced the level of oxidative stress indicator but at a significantly lower extent than that obtained by BCo. The SCo-induced reduction in MDA and POD was 17.4 % and 21.9 %, respectively, compared to 13.43 % in SOD. Similarly, treating potato leaves with different microelements markedly reduced the POD and SOD activities and MDA. The decrease was in the order of Se < B < Co < Ti, respectively. The maximum decline in POD (45.8 %), SOD (27.8 %), and MDA (39.4 %) was achieved by co-application of BCo with B which was statistically at par with Se for SOD and MDA.

**Table 8:** Effect of adding organic manures, microelements, and their interaction on peroxidase (POD), superoxidase dismutase (SOD) enzymes, and malondialdehyde (MDA) in leaves of potato plants grown on salt-affected soil for 75 days from planting.

		Peroxidase (Unit mg^−1^ protein)	Superoxide dismutase (Unit mg^−1^ protein)	Malondialdehyde (μmol.g^−1^ FWT)
Compost manures (Co)				
Control		2.71a ±0.033	2.65a ±0.021	5.80a ±0.069
Banana (BCo)		1.93c ± 0.074	2.12c ± 0.040	4.16c ± 0.127
Soybean (SCO)		2.25b ± 0.046	2.29b ± 0.029	4.76b ± 0.089
Foliar applications				
Control (C)		2.58a ±0.075	2.54a ±0.054	5.50a ±0.16
Boron (B)		2.09e ± 0.14	2.23e ± 0.081	4.50e ± 0.25
Selenium (Se)		2.16d ± 0.12	2.28d ± 0.087	4.67d ± 0.26
Cobalt (Co)		2.27c ± 0.12	2.33c ± 0.081	4.83c ± 0.25
Titanium (Ti)		2.38b ± 0.11	2.38b ± 0.087	5.01b ± 0.26
Interaction				
Control	**C**	2.88a ±0.02	2.76a ±0.019	6.16a ±0.038
	**B**	2.54e ± 0.011	2.54d ± 0.018	5.44e ± 0.052
	**Se**	2.62d ± 0.010	2.61c ± 0.016	5.64d ± 0.050
	**Co**	2.73c ± 0.013	2.63c ± 0.02	5.77c ± 0.055
	**Ti**	2.80b ± 0.02	2.70b ± 0.019	5.97b ± 0.060
BCo	**C**	2.39g ± 0.008	2.41f ± 0.016	5.06g ± 0.034
	**B**	1.56n ± 0.011	1.99k ± 0.015	3.73n ± 0.023
	**Se**	1.76 m ± 0.006	2.02k ± 0.016	3.85n ± 0.040
	**Co**	1.898l ± 0.011	2.07j ± 0.008	3.98 m ± 0.023
	**Ti**	2.03k ± 0.008	2.10j ± 0.016	4.16l ± 0.026
SCo	**C**	2.48f ± 0.018	2.46e ± 0.010	5.28f ± 0.051
	**B**	2.17i ± 0.008	2.17i ± 0.016	4.35k ± 0.027
	**Se**	2.09j ± 0.016	2.20i ± 0.008	4.53j ± 0.044
	**Co**	2.19i ± 0.008	2.28h ± 0.010	4.74i ± 0.027
	**Ti**	2.297h ± 0.010	2.34g ± 0.010	4.90h ± 0.046

Data represent the mean of three biological replicates ± SE. Means within a column followed by a different letter (s) are statistically different at a 0.05 probability level.

These results reveal the attenuative effects of the combined treatment of BCo and trace elements, particularly B, on the salinity-induced oxidative stress. MDA content is an important bio-indicator of plant oxidative damage [46]. It is the outcome of the ROS-induced lipid peroxidation of cellular membranes, which induces oxidative damage to cell components like membranes, nucleic acids, and proteins. Consequently, the significantly high MDA level in the salinity-stressed potato plants in the current experiment reflects the intensive oxidative stress they encounter. These results are consistent with those of [50,68]. The high MDA content in the salinity-stressed plants is attributed to its associated accumulation of toxic Na^+^ and Cl^−^ levels and lack of water availability, which could induce ROS production and consequently inhibit photosynthesis and protein synthesis [67].

Interestingly, the significant reductions in MDA levels in potato plants in response to the application of BCo and SCo and the tested elements reflect the efficient roles of these treatments in reducing the salinity-induced ROS and, thus, lowering MDA in potatoes [17,96]. The pronounced efficiency of the combined treatment of BCo and B in reducing the MDA may be also attributed to the role of B in the induction of the synthesis and accumulation of the non-antioxidant compounds [97]. Moreover, less accumulation of MDA will increase the Mg uptake and thereby chlorophyll content [98]. Such protective effects of the composts and microelement treatments against oxidative stress partially explain their positive impact on the growth and productivity of salt-stressed potato plants (Table 3, Table 4).

Under salinity stress, plants tend to activate their antioxidant enzymes as a part of their protective mechanisms to reduce salinity-induced ROS production and consequently minimize oxidative stress [14]. Therefore, along with their high level of MDA, the significantly high activities of POD and SOD in the salinity-stressed potato plants reflect the intensive oxidative stress they encounter. On the other hand, the significant reduction in the activities of these enzymes in response to the application of BCo and SCo and micronutrients revealed the ameliorative role of these treatments against the salinity-induced deteriorative impacts. Similar promotive effects of organic fertilizers on salinity-stressed plants have been reported [[99], [100], [101]]. Lastly, the co-incorporation of composts with microelements has proven beneficial in diminishing oxidative stress and increasing potato growth and productivity by restoring leaf nutrient status and chlorophyll content under salinity conditions.

### 3.6 Heat map and principal component analysis (PCA)

**Fig. 1:**
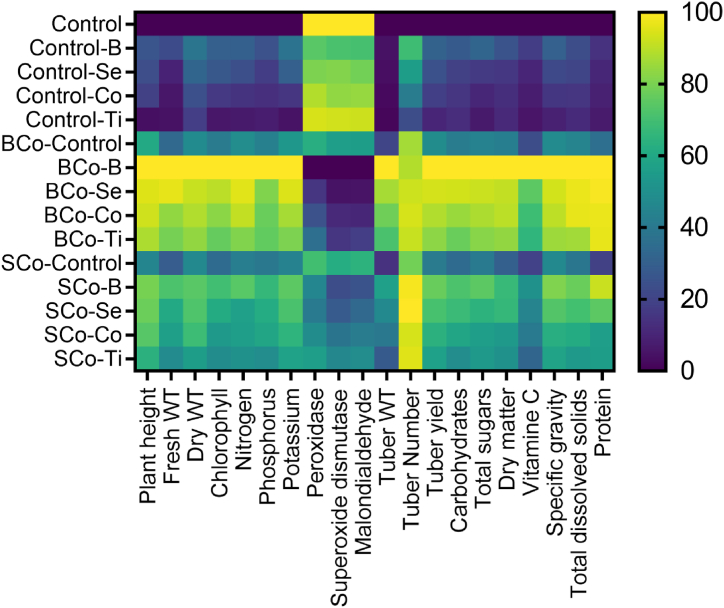
Heat map analysis for the vegetative, physiological characteristics, and yield components of salt-affected potato plants grown under combined addition of organic composts (control, banana compost: BCo, soybean compost: SCo) and microelements (control: C, boron: B, selenium: Se, cobalt: Co, and titanium: Ti) on the studied variables. Different treatments are control, control-B, control-Se, control-Co, control-Ti, BCo-control, BCo–B, BCo–Se, BCo–Co, BCo–Ti, SCo-control, SCo–B, SCo–Se, SCo–Co, and SCo–Ti).

The heat map presented in Fig. 1 summarizes overall assessments of the studied traits including plant height, fresh and dry weight, chlorophyll, NPK elements, antioxidant properties, and yield attributes of potato plants grown under saline soil and treated with BCo and SCo along with microelements. It discriminated the BCo treatment combined with B and Se from all other treatments. This distinction revealed the positive influence of such a combination in enhancing growth, physiological processes, and productivity outcomes of the potato plants.

**Fig. 2:**
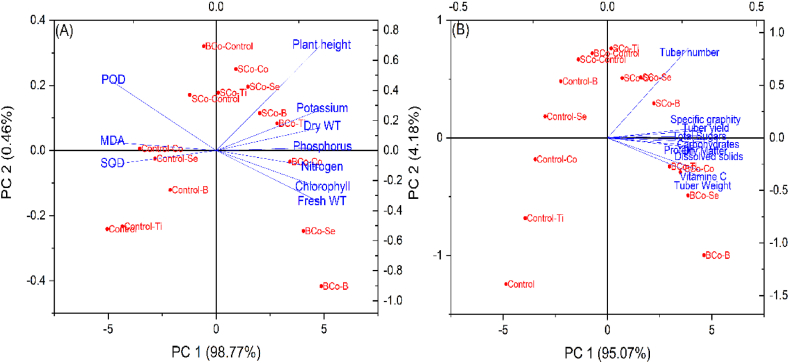
Principal component analysis (PCA) showing the effect of the combined addition of organic composts (Control, banana compost: BCo, soybean compost: SCo) and trace elements (control: C, boron: B, selenium: Se, cobalt: Co, and titanium: Ti) on the studied variables. Different treatments are control, control-B, control-Se, control-Co, control-Ti, BCo-control, BCo–B, BCo–Se, BCo–Co, BCo–Ti, SCo-control, SCo–B, SCo–Se, SCo–Co, SCo–Ti. **(A)**: Principal component analysis of different treatments, plant growth traits (plant height, fresh weight, and dry weight), chlorophyll content, NPK accumulation, and antioxidant properties (POD: peroxidase enzyme, SOD: superoxide dismutase enzyme, MDA: malonaldehyde). **(B)**: Principal component analysis of different treatments, potato tubers yield attributes (tuber weight, tuber number, and tuber yield), and quality parameters (total carbohydrates, total sugars, dry matter, Vitamin C, specific gravity, and dissolved solids).

PCA approach was also carried out to represent the differences in morpho-physiological traits, antioxidant properties, and tuber productivity indicators of salinity-stressed potato treated with BCo and SCo along with microelements (Fig. 2A&B). The two principal components (PC1 and PC2) covered 100 % of the total variance. PC1 had strong loadings to growth parameters, NPK elements, chlorophyll content, tubers yield, and biochemical characteristics, which were greatly affected by organic manures and trace elements. Among these, BCo combined with B or Se was the most effective treatment to improve potato productivity under stress. On the other hand, PC2 was positively loaded by MDA content, POD, and SOD enzymes, which were increased by salinity treatment and decreased by co-application treatments. PCA analysis also revealed that growth traits, chlorophyll content, and NPK elements were positively correlated, while strong negative correlations were observed among oxidative stress markers (MDA content and activities of POD and SOD enzymes) and the rest of the tested traits. It also showed strong positive correlations among all yield and agronomic attributes. These results ultimately indicated the vital roles of organic manures and microelements supplementation in enhancing plant growth and leaf nutrient status, which resulted in increased yield and productivity of potato tubers under salinity conditions.

## 4 Conclusion

Developing effective strategies to mitigate salinity stress is essential for sustaining potato growth and yield. The applying of banana and soybean composts which are eco-friendly and abundant along with microelements (B, Se, Co, and Ti) significantly increased growth parameters, nutrient status, and productivity of potato plants under saline conditions. The highest relative increase in the tuber yield was exhibited by banana compost supplemented with either B or Se. These positive responses were associated with a reduction in salinity-induced oxidative stress in potato plants. Therefore, the co-application of organic composts with trace elements can enhance potato productivity in saline environments while promoting the reuse of agricultural waste. It is also considered a promising strategy and future recommendation for developing sustainable and cost-effective organic fertilizers. We also still need to get deeper into the physiological and molecular mechanisms by which these amendments improve potato tolerance to salinity stress.

## Funding source

This study was supported by the Researchers Supporting Project number (RSP2024R347), 10.13039/501100002383King Saud University, Riyadh, Saudi Arabia.

## Data availability statement

All the data supporting the results of this study are included in this article.

## CRediT authorship contribution statement

**Ayman M. El-Ghamry:** Writing – review & editing, Writing – original draft, Supervision, Software, Project administration, Methodology, Investigation, Formal analysis, Data curation, Conceptualization. **Mohamed A. El-Sherpiny:** Writing – review & editing, Writing – original draft, Visualization, Validation, Supervision, Software, Project administration, Methodology, Investigation, Funding acquisition, Formal analysis, Data curation, Conceptualization. **Abd-Elbaset A. Alkharpotly:** Writing – review & editing, Writing – original draft, Visualization, Validation, Supervision, Software, Project administration, Investigation, Formal analysis, Data curation, Conceptualization. **Dina A. Ghazi:** Writing – review & editing, Writing – original draft, Visualization, Validation, Supervision, Software, Project administration, Investigation, Formal analysis, Data curation, Conceptualization. **Amal A. Helmy:** Writing – review & editing, Writing – original draft, Visualization, Validation, Supervision, Software, Project administration, Investigation, Formal analysis, Data curation, Conceptualization. **Manzer H. Siddiqui:** Writing – review & editing, Funding acquisition. **Mohammad Pessarakli:** Writing – review & editing. **Mohammad Anwar Hossain:** Writing – review & editing. **Eman M. Elghareeb:** Writing – review & editing, Writing – original draft, Visualization, Validation, Supervision, Software, Project administration, Methodology, Investigation, Formal analysis, Data curation, Conceptualization.

## Declaration of competing interest

The authors declare that they have no known competing financial interests or personal relationships that could have appeared to influence the work reported in this paper.
